# “And this is the life jacket, the lifeline they’ve been wanting”: Participant perspectives on navigating challenges and successes of prescribed safer supply

**DOI:** 10.1371/journal.pone.0299801

**Published:** 2024-03-22

**Authors:** Nancy Henderson, John Marris, Kirsten Woodend

**Affiliations:** 1 School of Nursing, University of Victoria, Victoria, BC, Canada; 2 John Marris Consulting, Peterborough, ON, Canada; 3 School of Nursing, Trent University, Peterborough, ON, Canada; University of Connecticut Health Center: UConn Health, UNITED STATES

## Abstract

**Background:**

In 2021, 43% of drug toxicity deaths in Ontario were reported by public health units serving medium-sized urban and rural communities. Safer supply programs (SSPs) have been primarily established in large urban centres. Given this, the current study is based on an evaluation of a SSP based in a medium-sized urban centre with a large catchment area that includes rural and Indigenous communities. The aim of this research paper is to understand the challenges and successes of the nurse practitioner-led SSP from the perspective of program participants.

**Methods:**

Interpretive description was used to understand the experiences of 14 participants accessing a SSP. Each participant was interviewed using a semi-structured approach, and 13 of the interviewees also completed surveys accessed through Qualtrics. An iterative process using NVivo software was used to code interviews, and a constant comparative data analysis approach was used to refine and categorize codes to themes.

**Findings:**

Three overarching themes were the result of this analysis: feeling better, renewed hope, and safety. These three themes capture the experiences of participants in the SSP, including both the challenges and successes they faced.

**Conclusion:**

The findings and subsequent discussion focus on both the key best practices of the program, and areas for future development and improvement. Despite barriers to services, prescribed SSPs are improving the lives of people who use drugs, and the current outcomes align with reports and evaluations from other SSPs across Canada.

## Introduction

In Canada, 38,514 people died due to opioid-related drug toxicity between January 2016 and March 2023 [1]. Twenty-one people die every day [1]. Drug toxicity deaths in the Canadian province of Ontario increased by 50% between 2019 and 2020, and numbers have remained high since, with 2,501 people dying in 2022 [1]. While deaths are prevalent in large urban centres, there is an emerging and notable concern related to the increase in deaths in medium-sized communities [2]. In 2021, 43% of opioid-related toxicity deaths in Ontario were reported by public health units serving medium-sized urban communities (1,238 of the 2,853 deaths): The rate of deaths per 100,000 people ranged from 6.3 in southern Ontario to 78.8 in northern Ontario where the percent case range has increased by 82% since 2020 [2].

The increase in people dying due to drug toxicity is directly related to the disruption to the unregulated drug supply which has rendered it adulterated, unpredictable, and toxic [3]. The adulteration of the drug supply, first with fentanyl and its analogues, and now with novel substances such as etizolam and xylazine, has increased deaths and has further complicated the associated crisis response in Canada. Deaths of this magnitude due to any other cause would be met with a robust public health response, yet we have not seen that for the drug toxicity crisis that was declared in British Columbia in 2016 [4, 5]. If the drug toxicity crisis received the same robust and organized public health response that was granted to the COVID-19 pandemic, we would see well supported and sufficiently funded innovative ideas and interdisciplinary collaborations, yet money and discretion to act have been notoriously denied to this crisis, especially for harm reduction responses [4]. Central to this lack of public health response is the stigmatization, criminalization, and marginalization of people who use drugs (PWUD) that is so deeply rooted in our systems, including the health care system [4, 6–8].

In part due to low retention rates and negative side effects in traditional treatment programs such as opioid agonist therapy [OAT; 9, 10] and the need to directly address the source of the drug toxicity crisis with a safer supply of drugs [11], a small number of pilot safer supply programs (SSP) have been funded by the federal government. When the tools available to combat the drug toxicity crisis are not working adequately, it is a fundamental requirement for governments and service providers to address this failure with innovative models to stop the unnecessary deaths [12]. The concept of safer supply is one of these innovative models. The original conception of *safe supply* was published in a 2019 guidance document by the Canadian Association of People Who Use Drugs. They called for the provision of a safe supply of all traditionally illicit drugs that would elicit euphoria and come from a legal and regulated market [13]. From this, the SSPs that are currently being funded by Health Canada are a medicalized, prescriber-based adaptation of this concept. These prescribed SSPs are rooted in harm reduction philosophy and provide participants who are at the highest risk of unregulated drug-related harms with pharmaceutical-grade medications of known quality and composition to replace the unpredictable and toxic street supply of drugs [13–16].

SSPs have been primarily established in large urban centres [17], although there is an increasing need to provide services to PWUD in small and medium-sized communities [18]. Given this, the current study is based on an evaluation of a SSP located in a medium-sized urban centre in Ontario with a large catchment area that includes rural and Indigenous communities. SSP participants are typically prescribed a combination of a long-acting opioid such as slow-release oral morphine (SROM; Kadian®) and/or methadone, along with an immediate-released opioid such as hydromorphone (Dilaudid®). The reasons for prescribing a long-acting opioid alongside an immediate-release opioid include helping to manage withdrawal symptoms and helping to increase the morphine equivalent dose to meet the recipients daily opioid needs since hydromorphone on its own is often ineffective at replacing the current street supply [19]. Some SSPs also offer pharmaceutical-grade replacements for stimulants, such as methylphenidate (Ritalin®); however, at the time of interviews, the SSP of this study was not. Participants typically receive their prescription daily at a pharmacy. They take their long-acting opioid witnessed at the pharmacy, and they get their immediate-release opioid tablets to take away with them. The immediate-release opioid tablets can be swallowed, snorted, or injected, depending on the decision made by the participant. Discussions about the route of ingestion are typical within the SSP and safety is regularly addressed by the SSP team. To further reduce harms, this SSP offered access to and training on harm reduction supplies, including Sterifilt+® filters (Apothicom, Paris, France) which provides those who choose to inject with another option for filtering their immediate-release opioid tablets [20, 21].

This pilot SSP is a nurse practitioner-led program that was developed based on extensive consultation with the local community of PWUD. Including PWUD in all aspects of SSP development, from design to implementation to evaluation, is crucial to the effective delivery of care [14, 22–24]. The process of holding interviews, focus groups, and advisory committee meetings led to the development of a SSP that, where possible, exists by and for the community of PWUD. These foundational relationships led to the development of a SSP community, which is an important success of the program that is critical for both participants and staff. The interviews with ordering and service providers along with the focus groups with PWUD were conducted in 2021 and they provided ideas and concepts about what the community wanted to see and how they wanted to benefit from a novel SSP. On a program level, these included access to primary care; community outreach to meet people where they are; options and choices in programming; respectful, holistic, individualized care; and opportunities to make and meet personal goals [25]. On a systems level, they asked for the involvement of people who use drugs; community education to combat stigmatization and criminalization; incorporation of relational, trauma-informed, culturally-specific programming; evidence creation to change drug policy; and increased access to basic needs, namely housing [25]. PWUD and those who offered services to them wanted to see SSP participants treated and cared for in ways that would be different from what they were accustomed to receiving from traditional health care and social services. The advisory committee was involved in the decision to offer Sterifilt+ filters; the drafting of policies, procedures, and evaluation documents; and while they had ideas about what medications ought to be available to SSP participants, this decision was ultimately made by the SSP prescriber, within the limitations of the Ontario Drug Benefit Formulary. The goal of this harm reduction-based program is to reduce the risk of drug toxicity deaths for its participants and provide low-barrier access to a safer alternative for people through relationship-based health care. This SSP also offers wraparound support, including primary health care, nursing care, case management, social support, and peer support.

The first participants were recruited in the Spring of 2022. Eligibility included people diagnosed with opioid use disorder [26] and who were deemed to be at high risk of drug-related harms or death. Risk rating was complex and multi-factorial, including the daily use of street-acquired opioids as well as individual impacts related to both social determinants of health (i.e., housing, income, social inclusion, and access to services including health care) and structural determinants of health (i.e., racism, gender oppression, and criminalization). There are currently no participants who are under the age of 19 in the program. At the time of interviewing, the program was reporting a 95% retention rate of participants.

A robust evaluation approach was developed for the pilot program to meet the requirements of the program’s funder and contribute to the broader development of knowledge about safer supply. While the full evaluation includes interviews and surveys with SSP participants, staff, and partners, the research tools contributing to this study included semi-structured interviews with SSP participants conducted seven months after the official launch of the program and detailed surveys of participants’ experiences over time. The rapid timeline for this assessment was beneficial since the findings have been used by the SSP staff for improving programming to better meet the needs of SSP participants and the local community of PWUD. For example, requests by participants for increased engagement led to the development of a clinic-based drop-in art program and a community-based garden of medicines and food that is collaboratively maintained by SSP staff and participants. The aim of this study was to understand the challenges and successes of this nurse practitioner-led SSP from the perspective of program participants.

## Methods

Interpretive description, a qualitative approach, was used to understand the experiences of participants accessing the SSP. This research methodology was developed to investigate patterns in human experience and use the information to increase knowledge and understanding that is applicable to practice and participant care [27]. Convenience sampling was used to recruit participants who were available at the time of interviewing. This method was chosen as it elicits varied perspectives and experiences of the SSP. The participants were interviewed using an interview guide and a semi-structured interview approach, and questions had associated prompts for clarification if necessary. The questions and associated prompts were developed based on consultations with the advisory committee and responses from staff during their interviews as part of the broader SSP evaluation. Examples of SSP participant questions included, “What would you like the general public to know about safer supply programs?”, “Can you tell me about how it has been getting to your appointments?”, “In what ways has being a part of the program taken up your time?”, and “Do you have any ideas for improving the program?” (see S1 File for a full list of questions). The interviewer made choices in real time about which subsequent questions to ask based on participant responses, but the overall structure was maintained throughout the interview process.

### Sampling

Interviews were conducted at the SSP clinic, a location that participants were familiar with. Participants were informed about taking part in an interview through in-person recruitment by two members of the SSP team who were identified through ethics as peer research assistants (PRAs). The interviewer (JM), the SSP’s external evaluator who was not known to the participants, was based at the clinic during the SSP’s clinical hours between November 17, 2022, and December 16, 2022. After their regular clinic visit, participants were introduced to the interviewer and aims of the research by the PRAs and offered the opportunity to take part in an interview.

Interviews were conducted in a private room. Participants were given the option of having a PRA present in the interview for additional support–one participant chose this option. Prior to providing informed written consent, the interviewer (JM) went over the letter of information and consent form with each participant, and all questions were answered to the participant’s satisfaction. Interviews took between 20 and 40 minutes and they were audio recorded for transcription purposes. Field notes were made by the interviewer to highlight key moments in the interviews and to aid in the analysis process. Participants were given a $25 honorarium to acknowledge their time, expertise, and contribution to the research. In addition to the interviews, data for this report was drawn from surveys completed by participants at baseline and then four months later, between October 2022 and February 2023. Again, informed written consent was provided as previously described. Surveys were completed using Qualtrics and participants were compensated $40 for their time.

Fourteen SSP participants were interviewed and 13 completed surveys: This number was sufficient to permit the authors to reach a coherent response [27]. The concept of coherent response is key to the interpretive description methodology and highlights the purpose of the research which is to provide a meaningful description of this phenomenon in a way that is applicable to practice and participant care [27]. For this study, after 14 interviews the researchers believed they could meaningfully describe the SSP in a way that could assist readers in creating a similar program with guidance on challenges and successes that would be relevant to consider for effective participant care. Four people declined participation in interviews, primarily due to lack of available time. No participants withdrew from the study.

### Inclusion criteria

Participants in the study met criteria to be enrolled in the SSP, which includes people diagnosed with opioid use disorder [26] and who were deemed to be at high risk of drug-related harms or death. Participants were able to give written informed consent and were fluent in English. There were no specific exclusion criteria.

### Ethics

This study was approved by the Trent University Research Ethics Board (REB File # 28007). Written informed consent was provided by each research participant prior to completing both their interview and survey.

### Analysis

A colleague of JM, who was listed as a co-investigator through ethics, transcribed the interviews verbatim, and JM reviewed them for accuracy. Each author (NH, JM, and KW) took an iterative approach to assigning initial codes to the interviews. This included uploading transcribed interviews to NVivo software with each author individually coding the 14 interviews. After this initial coding, the report authors compared the results of their individual coding to refine codes and discuss themes. Examples of initial codes included pain, collective trauma, no judgement, staff relationships, pharmacy experiences, time, basic needs, access to health care, and illicit drug market. Constant comparative data analysis was used as the codes were further analyzed and categorized into the three final themes that were deemed the most encompassing of the initial codes. In keeping with interpretive descriptive methodology, the application of the findings to practice and the potential for application of results was of key importance in this process [27]. The findings described below emerged from this collaborative process.

### Findings

Of the 14 interview participants, 13 answered the survey (Table 1). The majority of participants identified as white (71%), male (54%), with a mean age of 39 years (IQR 27–59). Most participants reported being unhoused (85%), with 75% stating their housing situation either stayed the same or got worse in the previous four months. Thirty-nine percent of participants reported having difficulty accessing food, while 77% reported having difficulty accessing basic needs. All participants who were using street-acquired fentanyl when they started in the SSP reported that they either decreased (85%) or stopped (8%) their use, and 92% reported their stimulant use either decreased (62%) or stayed the same (31%) in the previous four months. People reported decreased involvement in criminalized activities (54%) and decreased overdoses (94%) in the previous four months.

**Table 1:** Participant sociodemographic characteristics (n = 13)^a^.

	n	%
Gender		
Male	7	53.8
Female	5	38.5
Non-binary/2-Spirit	1	7.7
Ethnic Group^b^		
White	10	71.4
Indigenous	4	28.6
Housing		
Apartment, house, condo (rented or owned)	2	15.4
Shelter	4	30.7
Couch surfing	2	15.4
Hotel or room (weekly or nightly rental)	2	15.4
Sleeping rough, tent	2	15.4
Sleeping in car	1	7.7
Did your housing situation^c^		
Get worse	4	33.3
Stay the same	5	41.7
Improve	3	25.0
Difficulty accessing food		
No	8	61.5
Yes	5	38.5
Difficulty accessing basic needs		
No	3	23.1
Yes	10	76.9
Use of fentanyl since starting SSP		
Stopped	1	7.7
Decreased	11	84.6
Stayed the same	0	0
Increased	0	0
Was not using fentanyl (using street-acquired pills)	1	7.7
Use of stimulants since starting SSP		
Stopped	0	0
Decreased	8	61.5
Stayed the same	4	30.8
Increased	1	7.7
Was not using	0	0
Involvement in criminalized activities		
No	8	61.5
Yes	5	38.5
Involvement in criminalized activities has		
Never involved	3	23.1
Decreased	7	53.8
Stayed the same	3	23.1
Increased	0	0
Overdose in the past 4 months		
No	12	92.3
Yes	1	7.7
Age	Mean	Range
	38.9	27–59

^a^ One interview participant opted out of the survey portion of the evaluation.

^b^ Able to choose all that apply.

^c^ One person did not answer this question.

Three overarching themes were the result of this analysis: feeling better, renewed hope, and safety. These three themes capture the broad sweep of participants’ experiences in the SSP, including both the successes and challenges they faced. In line with an interpretive description methodology, this focuses the findings and subsequent discussion on both the key best practices of the program, and areas for future development and improvement.

### Feeling better

All participants relied on the unpredictable and toxic street supply of drugs prior to being connected with the SSP. Many felt that before joining the SSP they had no options left to create positive changes in their lives. They spoke about being tired of the daily struggles surrounding poverty and drug use and needing something to assist them in exiting this cycle.

[A] lot of the folks are tired. Tired of instability, tired of committing crimes. I’m telling you this. People think people like this lifestyle, but they don’t. They’re sick of it. And this [safer supply] is the life jacket, the lifeline they’ve been wanting. And you could see from the amount of people that are dying to apply, that are on the waiting list. A lot of people are just sick of living on the street, not knowing what they’re going to eat.

*Alex*

Participants spoke about the initial benefits of having access to a safer supply of a medication that could provide the desired euphoria and help them to manage their withdrawal and related sickness:

[I]t’s giving me an opportunity to use something that’s safer than street drugs, heroin or fentanyl or whatever. And it’s still… you’re still getting what you’re looking for. You’re still getting the high, or you’re still getting the help with withdrawal…. You’re not sick all the time.

*Taylor*

Having access to a safer supply of opioids and the reduction in sickness due to opioid withdrawal were not the sole benefits of the program. Nine of the 14 interviewees reported that they experience chronic pain, yet none of them were being treated for their pain in the period immediately prior to starting in the SSP. Some participants believe that the drug toxicity crisis that we are immersed in today would not be the same if it were not for the crackdown on opioid prescribing that led to physicians deprescribing to pain patients.

A lot of people that you see in the street right now doing fentanyl were the same people…. They had good, alright jobs. They had somewhat of a stable setting. Then the same doctors cut them off. And then what did they do? They started with heroin. And heroin disappeared and then they’re on to fentanyl. But I guarantee you if this program was there 10 years ago, we would not have this epidemic that we’re seeing today.

*Alex*

The deprescribing was directly related to the stigma and judgment surrounding opioid use disorder. Through the SSP, the needs of participants related to pain are being met with dignity and respect.

I wanted to not feel in pain anymore, not be judged by doctors saying my back doesn’t hurt and my arthritis ain’t that bad. Here I’m not judged about it. I can take my painkillers when I’m hurting, as needed. They understand….

*Quin*

With access to the SSP comes access to primary health care, including referrals to social services and specialized clinics. All participants spoke positively about their primary care experiences delivered by an NP (sometimes referred to as their doctor). They spoke about having “a chance to see a proper doctor” (*Quin*), having “[s]omeone to ask [and] a doctor to do something” (*Max*), being “treated like a human” (*Quin*), and feeling “comfortable” (*Sam*). They acknowledged the efforts made by the SSP team to arrange for tests, referrals, and surgeries. Having a safer supply in addition to robust and thorough primary health care and social support has led to the experience of wellness, as explained by this participant’s experience of receiving comprehensive care:

“And that [primary health care] on its own was, like, one of the good things that help, like, make everything so much better and easier, is to have the help you need. The positiveness. And I’m not just going to tackle one problem. It’s being able to tackle all the problems that basically feed into the one problem.”

*Quin*

Though this was not a universal position, some participants noted that attending the clinic for appointments and visiting the pharmacy for the dispensing of medications provided positive structure to their lives, which added to the sense of feeling better: “So just going to get my drink, and that being important. And having something to do in a day, right? Structure, I guess. Schedule. Planning” (*Max)*. However, getting to the pharmacy daily is not an easy task for all SSP participants, especially for those who face barriers and limitations due to their geographical location and the lack of pharmacies in some rural and Indigenous communities:

It takes me about 40 minutes to come into town. That is an issue with me with the program, actually. I can’t get carries and it costs me $20 a day to come into town every day to get my prescription filled…. Make the carry more of an option for people who are in like, doing well on the program like me, like I haven’t used, been doing fentanyl for what, like in months, right? That’s the whole purpose.

*Geri*

Access to carries, which is a prescription for multiple days’ worth of medication instead of only receiving enough medication to get them through that specific day, was requested by a number of participants who stated they were doing well based on program goals. Having access to carries is seen as a marker of success and was believed to be a service that would further improve their life and sense of feeling better.

The general sense of wellbeing that can be achieved through access to the SSP was summarized by one participant: “I feel better about myself. I just all around feel better, you know what I mean? I’m not chasing the high around. I don’t have body aches and body pains like I used to, and everything else, right” (*Sam)*.

### Renewed hope

Because people are feeling better, there was a sense of renewed hope that resonated through the 14 interviews. For this participant, they felt that PWUD had lost all hope until the realization that there was a new program in town that they could join and potentially change the trajectory of their life.

There’s been a renewed spirit I think for people who say, ‘Hey, there’s another program. There’s another way.’ Look at it changing the lives of the people on it. And then also look at [us], like we’re completely different people. It’s renewed hope, which was almost lost.

*Jack*

Beyond hope for simply staying alive, participants were able to see and feel the changes in their lives, which led to newfound optimism, inspiration, and motivation. They are witnessing change at a rapid rate, something that they had not expected and that is pushing them forward in new ways.

Yeah everything changed tremendously. My life switched right around. I’m more motivated. I feel less miserable about life…. It’s like there’s a light at the end of the tunnel. You get more optimistic because you can actually see a change. You see progression. When you’ve a goal in mind, and you actually see something transforming, it’s inspiring. It makes you feel good. It keeps the motivation going.

*Quin*

For some participants, the SSP clinic is a place they look forward to going: “[I]t’s the high point of my life right now. I didn’t believe I had any hope in hell of having any future before this” (*Bobbi*). The SSP clinic is providing participants with a space to congregate and form connections: “The social aspect of being able to come down and talk to people” (*Max*).

Renewed hope was not always related to the SSP and its structure. It also came through accessing paid work, and the hope that accompanies a sense of stability and a feeling of optimism for the future. This involved the participant building their own structure and schedule, something many were not able to do when involved in the cycle of procuring and using drugs: “I’ve been able to get up and go to work. I feel better about myself. I feel like I’ve accomplished more. Every day I wake up I have something to look forward to” (*Parker)*. Having a sense of purpose and accomplishment was felt by participants as they found things to occupy their time. Being freed of the burden of participating in the daily hustle allowed them to seek other opportunities, such as work and drop-in programming. One participant noted that they had succeeded in attaining 12 hours of their day back. Participants also reflected on how participation in the SSP living experience advisory committee and SSP research (surveys and interviews) was of great importance to them.

The renewed hope was not only centered around themselves and their own lives. Participants were hopeful for their community, and they were optimistic that the SSP is making a difference in decreasing the risk of death for those who are in the program: “People don’t understand, like, what a big help this is. At least half the friends that I have right now are alive still. And I think it’s because of this program” (*Jack)*. Participants also pointed out how this program benefits the whole community through a reduction in crime. In one interview, the participant broke down how to renew hope within the broader community, especially among people who only care about money and who are not compassionate about, or understanding of, the barriers and struggles faced by PWUD who are stigmatized and criminalized.

[O]kay, sure, you don’t care about the person. But imagine how much harm they do to the community while they’re still alive. So, like, do you not care about the jewelry store that’s getting robbed, the pharmacies that are getting robbed, the convenience store that’s getting… the cost of all that, you know? This [program] is way cheaper than all of that. And when you show them that, that’s when they understand that. They care about the money and their car not getting broken into.

*Alex*

### Safety

For many participants, the fear of drug toxicity harm or death and the trauma of witnessing their friends die was part of their motivation for joining the SSP.

I was worried. Like, everybody’s overdosing, and people are dying and getting drugs that they’re not thinking they’re doing. And I was using fentanyl. I never had an overdose or anything, but I was worried about if I did, I wouldn’t wake up. That was the main reason. I just didn’t want to die.

*Charlie*

Many people have died due to the drug toxicity crisis [1], which participants noted had led to grief and loss. One participant spoke about how unexpected and devastating this has been to the community.

I go to the [drop-in centre] almost every day. And not necessarily just to eat or to whatever. Just to drop in and say ‘hi’ because so many people have died. It’s just incredible to see how, like, people have passed away. You never would have expected it…. I’ve lost so many friends due to the overdose problem.

*Jaime*

People seek safety, and in some ways, they are finding it through their participation in the SSP. One participant described how they have been able to eliminate drug toxicity events (referred to here as overdoses or ODs) from their life by having access to a safer supply and therefore being able to reduce their use of the toxic street supply. This has led to a reduction in fear and a new feeling of safety.

I haven’t OD’d since I’ve been on [hydromorphone]. I used to OD quite constantly, but I haven’t OD’d since I’ve been on it. I don’t use as much. I don’t use as much, like I don’t need as much fentanyl as I used to. It just kind of takes the weight away from me, right.

*Sam*

Although SSP participants reported that they are not experiencing drug toxicity events when using their safer supply, some acknowledged that they continued to access the toxic street supply because the prescribed medications do not meet their needs. Several participants called for a wider range of opioids to be made available so they can completely avoid and remain safe from the toxic street supply: “I would prefer something that has more of a kick to it than Dilaudids® for the cravings and stuff like that, like a, like a fentanyl without the side… like the risks.” (*Charlie)*.

Diversion was not specifically asked about, yet the topic was raised by four participants who spoke about the sharing or selling of some of the safer supply medications to friends who do not have access to a safer supply and are therefore at risk of harm and death every time they use drugs. For some participants, the sharing of their safer supply was understood to be a way of increasing the safety of the broader community. One person explained that some hydromorphone tablets are shared with friends in the community who are struggling with pain or withdrawal, and in return, they can use the money they receive to access basic needs. Participants also spoke about how they are often in a difficult position due to the extremely limited numbers of people who are able to gain access to a safer supply when everyone around them needs access too.

Well, it helps people get off the drugs. And some people do stuff with their meds sometimes [divert], but they try not to spend that on other things. They try to get the stuff they need with it…. But people, like, try to help other people as well…. And there’s only a certain amount of people that can be on the program right now. And I would like a lot more people to be on it because they need to be on it.

*Shane*

In addition to feelings of increased safety through their safer supply prescription, participants are gaining safety from their experiences and relationships with the SSP prescriber and supporting staff members.

I feel safe, for sure. People are very understanding. They don’t make me wait. They’re not judgmental at all. And they actually see me as…they interact with me, they ask personal… they care to know about my life.

*Alex*

Another participant states, “And everybody treats me. They treat me with dignity, respect, and everything” (*Shane*). The therapeutic relationships built with staff are reminding the participants of what they deserve to experience in relationships. Experiencing these positive therapeutic relationships allows participants to be honest with health care providers, free from fear of negative consequences.

Having the SSP located within a primary care clinic was seen as beneficial to those who wanted to remain anonymous and free from judgment from the general population. This anonymity led to a sense of safety accessing urgently needed services.

Unlike going to the methadone clinic, everybody knows it’s a clinic. It’s one straight thing. And just from people and like everyone else around you to go there, you feel like that judgment. You feel like shit walking into it, right? It’s like going and standing in a food bank line. Here, you’re walking in through [the primary care clinic door]. Nobody on the outside is gonna know the difference…. Because people are judgmental, sad to say it.

*Quin*

Similarly, the experience of going to the pharmacy was, for the majority of participants, a positive experience, free from judgment and stigma. One participant described their experience of going to pick up their safer supply prescription as “[j]ust as if you’re going in there picking up cough medicine” (*Quin*). Again, there was safety in going unnoticed.

With safety came freedom and agency. One participant appreciated the fact that they could access daily take-home doses of their safer supply, which allowed them to consume their medication how they wanted and when they needed it: “[T]he fact that you get to use it at your own risk, your own pace, like you don’t have to be told when and where you cannot use it. You just don’t feel…it feels like you have more freedom” (*Parker)*. Once again, the feeling was not solely personal. Participants wanted to see their communities thrive, with safety being a community-based experience. They wanted to see an end to the cycle of unregulated drug use and the associated crime, both through the hustle of PWUD and the organized crime that controls the unregulated drug market.

It’s helping people and it’s a positive for the community and for people that aren’t even addicts. Like, it’s probably stopping a lot of crime. Like, it’s doing more than just helping us, it’s helping the community… and my family is gonna benefit from it.

*Charlie*

## Discussion

Interviews took place with 14 participants of a small SSP in a medium-sized urban centre with a large catchment area that includes rural and Indigenous communities. From these interviews, feeling better, renewed hope, and safety emerged as the key themes that highlighted the challenges and successes of this SSP. When analyzed, the words and ideas that came from the interviews can be used to both improve existing, and inform new, models of safer supply.

Drug toxicity deaths, commonly referred to as overdoses, were of great concern to participants upon entering this SSP. It is evident through the participant interviews and their reports of overall decreased use of fentanyl, use of stimulants, overdoses, and reliance on criminalized activities (Table 1), that having access to a safer supply is contributing to increased health, hope, and safety. Similar reports of SSP participation leading to decreased use of illicit drugs, risk of overdose [22, 28–32], and risk of criminalization [28, 29] have been found in other studies.

When looking at challenges, prescribed SSPs in Ontario are overly medicalized [33] with guidelines requiring participants to make frequent clinic visits for prescriptions [24, 34], participate in urine drug screening [16, 35], and go to the pharmacy daily for both witnessed long-acting medication ingestion [16, 24, 35] and immediate-release medication pick-up [34, 36]. While some participants were accepting of these requirements, the medicalization of SSPs is reported to be a barrier to attraction for PWUD due to their general mistrust in the health care system, based on decades of criminalization and stigmatization [17, 33]. Some participants reported that the structure prevented them from living their lives, accessing paid work, and visiting out of town family and friends.

In contrast, the overall structuring of the prescribed SSP was welcomed, and even enjoyed, by some participants as it allowed them to create a schedule and build safety and trust. Participants appreciated that even if they missed their appointment time, staff would make efforts to accommodate them. This was reported to be different from most other experiences within health care, including experiences with OAT where PWUD are often penalized for missed appointments. The new sense of purpose and accomplishment, and having things to occupy their time, allowed participants to thrive. Similar experiences have been reported by participants of another SSP where participants talked about developing a routine [23]. These contrasting experiences with medicalization and structure point to the need to implement alternative, innovative models of safer supply that would allow participants to have agency and autonomy over their lives. This could include looking at options for non-prescribed safer supply models, such as compassion clubs, where participants can access their safer supply without needing a prescription [28, 33, 35–38].

While some participants were satisfied with receiving prescribed hydromorphone tablets, this was not the case for all, as was echoed in another study where this was the only option [22]. Several participants noted the need for a wider range of opioids to be available through the SSP, including options that match the desired effect or euphoria of the street supply and that can be smoked. Despite calls on the Ontario government to expand available options [39], the only available immediate-release medication that is currently covered by the Ontario Drug Benefit Formulary and can come close to matching the tolerance of people who are reliant on street-acquired fentanyl is hydromorphone tablets. The street supply of drugs is reported to have become increasingly more potent and toxic, especially immediately prior to and since the onset of the COVID-19 pandemic [40, 41]. The currently available hydromorphone tablets often lack the ability to truly replace the individual’s use of street-acquired opioids [17] that has averaged between 3.6% and 8.2% fentanyl in Ontario over the past year [42]. Therefore, many people attending a SSP, that has standardized protocols with limitations and maximum doses of hydromorphone tablets [23], remain reliant on the street supply to meet their opioid tolerance needs. While participants of SSPs are partially protected from the risk of drug toxicity harms and death due to their access to a safer supply, their risk of death remains high every time they access and use the unregulated and toxic street supply of drugs. PWUD need options, including access to the right drug, in the right dose, through the right route, and at the right time [35].

Statistics related to the percentage of people with opioid use disorder who have access to a safer supply prescription in Ontario are not currently available; however, if we look to British Columbia as an exemplar, where safer supply prescribing is more prevalent, it is estimated that less than 5% of people with opioid use disorder have access to a safer supply prescription [43, 44]. This highlights the call from SSP participants in this study related to friends and family not having access, which has also been reported by other SSPs that state that they have so far been unable to meet the needs expressed by the community [16, 32]. In one report, participants spoke about the negative effects of having survivors guilt [28]. What is ultimately needed is easy and equitable access to a safer supply [14, 45]. There are challenges to scaling up prescribed SSPs as they exist today to serve all PWUD due to issues such as operational costs, staff shortages, structural inequities, limited access to desired medications, and overmedicalization of services [33]. This speaks to the need for a variety of SSP models, prescribed and non-prescribed, to reach everyone who needs it. Having multiple models and options would give PWUD agency and choice in accessing their safer supply, while leaving prescribed SSP spaces open to those who require more intensive support.

Although not directly asked in the interviews, nine of the 14 participants (64%) spoke about their experiences with chronic pain, often referring to the stigmatizing and judgemental practices of prescribers as they discontinued their opioid prescriptions without a realistic alternative plan. This is not a new narrative and it has been reported by participants of other SSPs [29]. After a period of what was considered excessive prescribing of opioids for chronic pain, there was a call for physicians to alter their practice, leading to mass deprescribing starting in 2010 [46] followed by a delisting of OxyContin® in 2012, both of which were not evidence-based practices [47]. These were in fact reactionary practices in response to the growing number of people dying from opioid-related toxicity [47, 48]. The deprescribing or sudden decrease in dose has been shown to increase the person’s risk of suicide, drug toxicity death, uncontrolled pain, and other adverse events [49, 50]. It is not merely a coincidence that the deprescribing of opioids coincided with an upward swing in the use of unregulated opioids, such as heroin and diverted or non-prescribed opioids [51], and a subsequent and predictable upward swing in drug toxicity deaths [46, 52]. PWUD, left to self-manage their chronic pain by accessing the street supply of drugs, were put at high-risk of drug related harms and death [53]: These risks need to be considered by prescribers when deciding on how to manage chronic pain with pharmaceutical-grade opioids for PWUD [54]. Although the drug toxicity crisis would not be declared for another six years, 2010 was the beginning of this crisis. Based on these findings, deprescribing is now considered to be an intervention with high-risk, and if undertaken, must be done at a very slow rate and from a person-centred perspective of care [49, 50, 55]. When looking at prescribed SSPs, they can be understood as efforts to re-prescribe a safer supply of opioids to those who have suffered the horrific outcomes of deprescribing over the past 13 years. Participants of this study spoke about how the re-prescribing of opioids led to them feeling better, heard, and understood, without fear of stigma and judgment. The access to pain management through this SSP based in a primary care clinic also provided a pathway to better overall physical and mental health as participants attended to chronic health conditions that had previously gone untreated, an experience that was shared by other SSP participants [29].

SSPs have an obligation to attend to the needs and goals of PWUD related to their drug use, including their wish to decrease use, abstain from use, continue to experience euphoric effects, or manage chronic pain [14, 16, 35, 56–58]. It must be noted that at its core, the SSP is fundamentally concerned with providing PWUD with a safer alternative to the toxic street supply of drugs. This must come with the SSP acknowledging that the goals of some PWUD include experiencing euphoria without the risks inherent to the street supply. While the SSP will support individual goals of reducing or stopping the use of street-acquired drugs, not all PWUD share these goals, and this must not impact their ability to join or continue to engage with the SSP. From there, it is the responsibility of the prescriber to offer individualized, person-centred care–the gold standard in health care practice today [59]. Person-centred care is the aim of most health care professionals in most health care settings, except, it would seem, for medicine practiced with PWUD, where the gold standard approach to treatment is the standardized addictions medicine model of OAT [14, 60, 61]. With OAT there is an expectation that PWUD adapt to a prescriber-determined model that typically includes frequent clinic visits with mandatory urine drug screening and contingency management strategies [62]. In contrast, the SSP is attempting to use an individualized, person-centred care approach, and this is evident through all three themes. The participants gained a sense of feeling better due to the options in health care and support available to them, renewed hope due to the newfound trust and respect they felt from the SSP staff, and a sense of safety in that they were finally being heard and cared for, without fear of negative repercussions. Participants highlighted how SSP staff went above and beyond to ensure they were receiving the best care possible, which was a common response from participants in other SSPs [23, 28, 29]. These are all necessary factors in program structure to promote access to, and trust in, the health care-based relationships and programming [16, 22, 24, 63].

The location of the SSP, being embedded in primary care, was a key contributor to participants noting overall benefits to their health and wellness, which was echoed in other studies [23, 28, 29]. In addition to health, participants gained a sense of safety due to the physical location of the clinic since people not associated with the program were not aware of why SSP participants were entering the primary care clinic. This was an experience that is quite different from another study where researchers spoke to participants of a SSP that was co-located in an overdose prevention site, resulting in conflicting outcomes of it providing both a low-threshold for, and a barrier to, engagement [22]. These varying participant responses highlight the need for different types of safer supply models, including locations that are both connected to, and separate from, services for people who continue to access the unregulated street supply of drugs [22].

When SSP participants knew where their next dose was coming from and that they had easy access to it, they felt a sense of comfort and safety. This was a feeling experienced by participants of other SSPs where they emphasized that they no longer had to participate in criminal activities to access drugs [17, 23, 28]. However, when participants were unsure of how they would get to the pharmacy the next day, that sense of safety was diminished. Limitations with hours, lack of a delivery model, and lack of access to carries to support those who face transportation barriers were reported as challenges with the SSP and pharmacies. Participants of other SSPs noted similar barriers, especially related to lack of access to carries [14, 22, 35] and living in small or rural communities [17, 18, 22]. This speaks to the need to maximize the flexibility of SSPs, especially surrounding carries, and recognise the critical role of location and access in the success of programs, particularly programs that serve rural and remote communities.

Participants reported receiving more from the SSP than just medication to replace the toxic street supply of drugs. By gaining access to a prescribed medication, participants could more easily attend to their basic needs, such as using their money to buy food, which arose through the themes of feeling better and safety. However, with most SSP participants unhoused and living in poverty, the SSP faces challenges in providing services that go beyond merely providing a safer supply. The improvement to SSP participants’ economic situation by way of accessing a safer supply was important as it allowed them to reduce the time they typically spent hustling for resources and drugs through criminalized and stigmatized activities, a theme commonly shared by SSP participants in other communities [22, 23, 28, 29]. Socio-structural barriers, including criminalization, poverty, and racism, are factors that frequently impact SSP participants [4]. While solving these systemic issues is beyond the scope of any SSP, program staff have an obligation to attend to the socio-structural determinants of health, with the goal of providing a renewed sense of freedom, hope, and agency, and an overall feeling of safety within the program.

Basic needs are not being met by participants simply by having access to a safer supply, and this was evident through participants’ discussions on their safety and the safety of the community of PWUD. For some SSP participants, they were required to sell or trade some of their hydromorphone tablets so they could attain, for example, necessary items or shelter. As was concluded in other studies, some SSP participants participated in the sharing or selling of some of their hydromorphone prescription in order to help their peers who do not have access to a safer supply and to help themselves in purchasing necessary supplies [23, 64, 65]. Witnessing their friends and family who are struggling with severe withdrawal and chronic pain symptoms without access to a safer supply places the small number of SSP participants in an extremely difficult situation needing to decide on how they might help their peers while also meeting their own needs. Although not directly asked in this study, there was no specific mention of SSP participants selling their hydromorphone tablets to people who were not known to them as existing opioid users or to youth [23, 64].

The SSP was developed and implemented in close consultation with the local community of PWUD through community focus groups and advisory committee meetings, which ensured the centring of lived and living experience voices. The sense of community that was clear through all three themes was an experience shared by participants of another SSP [23]. Though these community building activities address some of the needs of participants, individualized support, such as mental health and trauma therapy, are not part of this particular SSP. Access to these individualized supports would allow SSP participants to attend to personal needs and goals that cannot be adequately addressed through group and drop-in programming.

There were limitations to this study that need to be considered. Firstly, the findings of the study are based on the experiences of participants in a small SSP based in a medium-sized urban community in Ontario with a large catchment area that includes rural and Indigenous communities. Therefore, the findings may not reflect the experiences of participants from other communities or from varying models of safer supply. Secondly, this SSP predominantly offers immediate-release hydromorphone tablets, which is different from medications offered in some other communities in Ontario and across Canada. Thirdly, findings based on survey results have the limitation of recall bias and survey fatigue. In addition, the people who answered the survey and interview questions represent a sample of participants and should not be considered to be the opinion or experience of everyone in this SSP. Lastly, these findings represent a cohort of SSP participants who had been part of the program for a maximum of seven months at the time of interviewing. It is therefore likely that their experiences of, and reflections on, the program would differ from those who have been a part of a SSP for a longer period. Future studies should consider SSPs in other communities and interviews with people who have had the benefit of time.

## Conclusion

The findings of this study strongly support the need for prescribed SSPs through evidence of substantial successes and positive outcomes as reported by participants who are users of the services and therefore based on lived experience of the program. In addition to the successes, the participants provided open and honest reports of the barriers and challenges with this prescribed SSP. Despite barriers to services, PWUD who have gained access to this prescribed SSP are reporting improvements to their lives, and the outcomes of this SSP align with reports and evaluations from other SSPs across Canada. For those who have so far not been able to access a prescribed SSP or do not require the wraparound services embedded in a program like the one studied here, there is an urgent need to implement non-prescribed safer supply models to provide people with opportunities for improved health, hope, and safety.

## Supporting information

S1 File Participant interview guide. (TIF)
