# Supplementary figures and images for: “And this is the life jacket, the lifeline they’ve been wanting”: Participant perspectives on navigating challenges and successes of prescribed safer supply
Source: PLoS One. 2024 Mar 22;19(3):e0299801. doi: 10.1371/journal.pone.0299801 (PMC10959334; doi:10.1371/journal.pone.0299801)

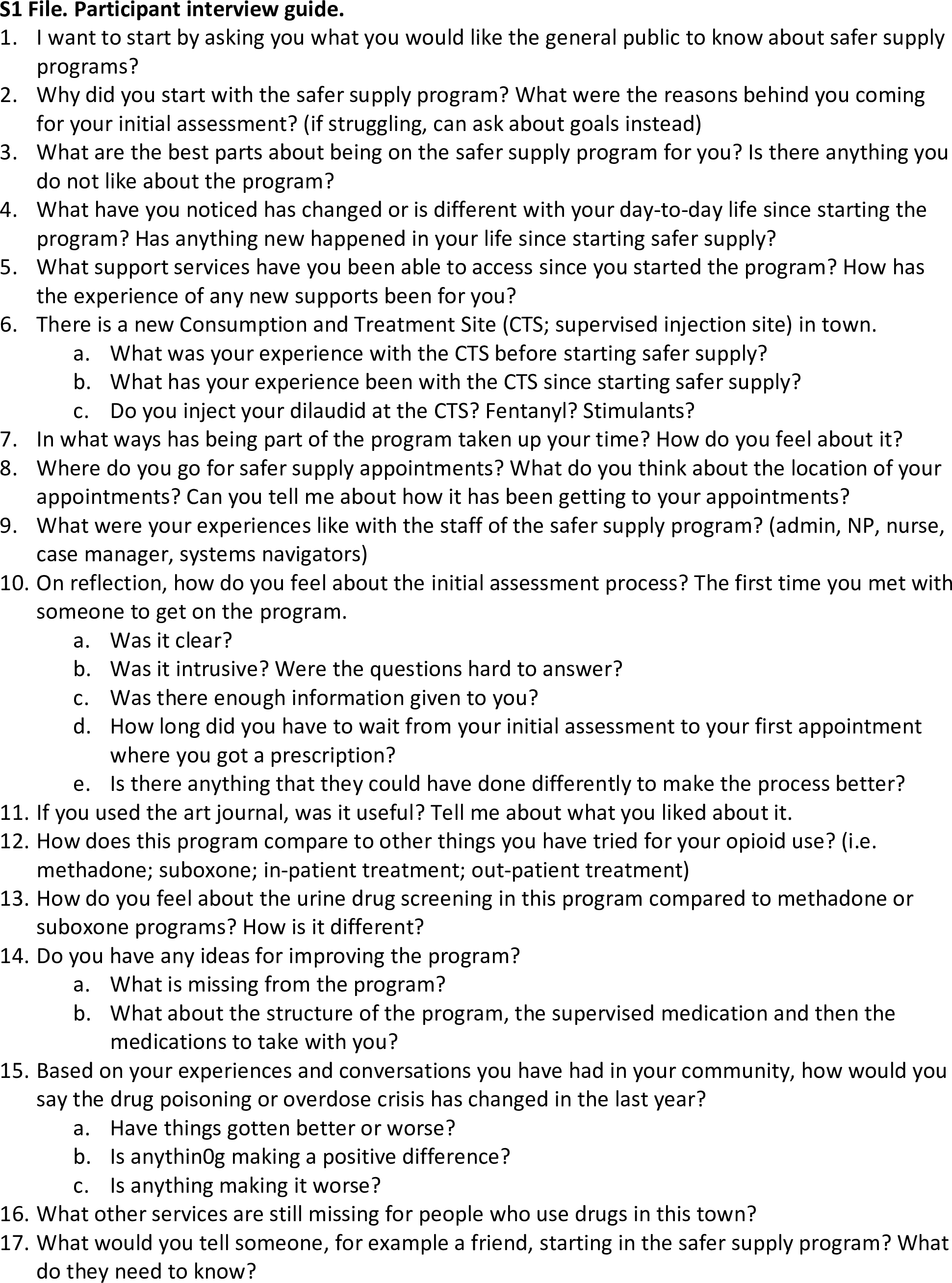

Supplement: S1 File — (TIF) [file pone.0299801.s001.tif]
